# Supplementary material for: Massive and parallel expression profiling using microarrayed single-cell sequencing
Source: Nat Commun. 2016 Oct 14;7:13182. doi: 10.1038/ncomms13182 (PMC5067491; doi:10.1038/ncomms13182)
Supplement: Supplementary Information — Supplementary Figures 1-8, Supplementary Methods and Supplementary References [file ncomms13182-s1.pdf]

Supplementary Figures

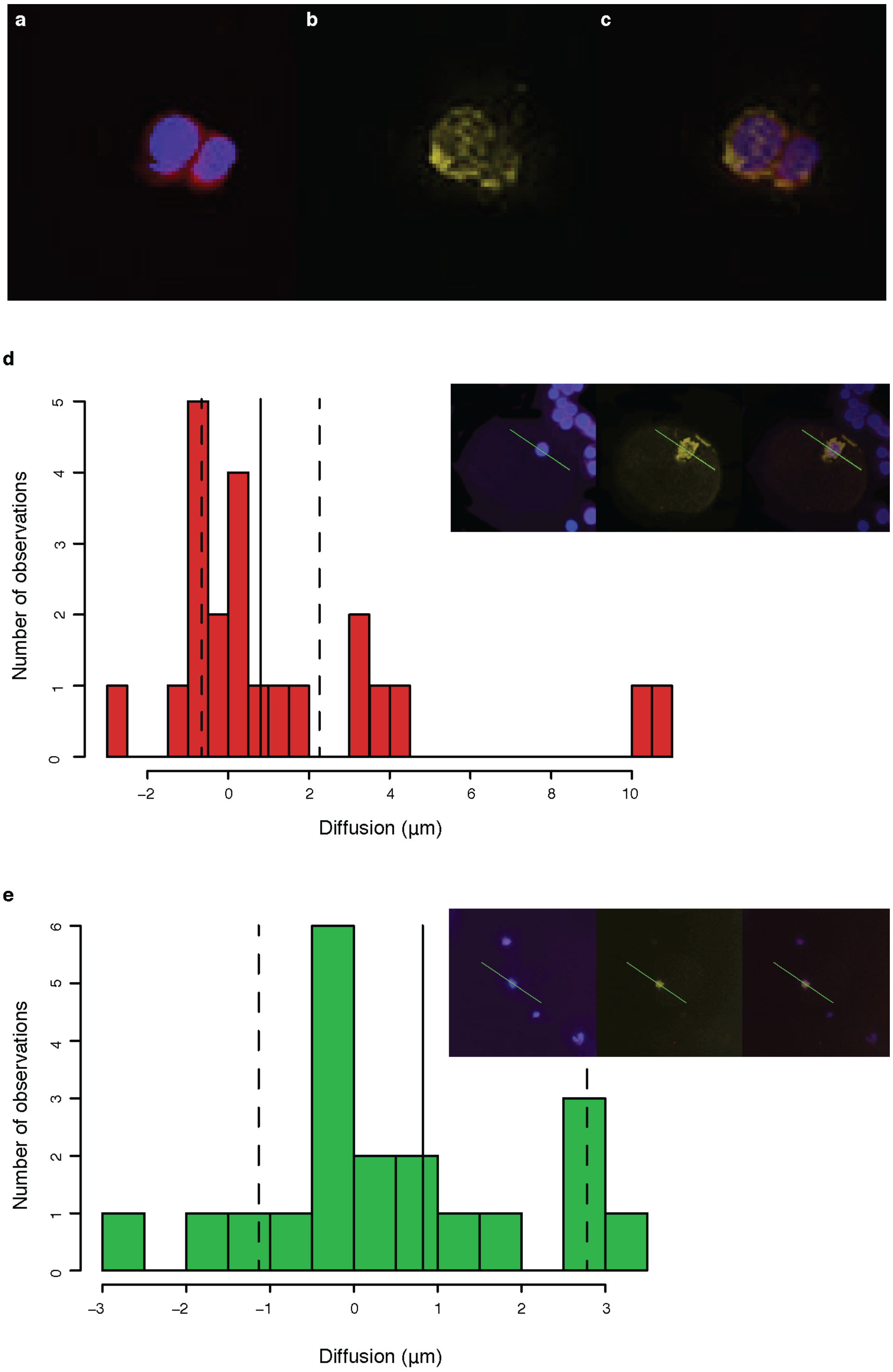

Supplementary Figure 1. Diffusion of MCF-7 and 3T3 cells. (a) High-resolution image (40x): DAPI and Cy5-CellTracker™ stained MCF-7 cells. (b) Corresponding high-resolution (40x) Cy3-signal after cDNA synthesis and tissue removal. (c) Overlay of images in (a) and (b). (d) Histogram of cDNA signal diffusion and images of MCF-7 cells. (c) Histogram of cDNA signal diffusion and images of 3T3 cells. Dashed line represents the standard deviation while the solid line represents the mean.

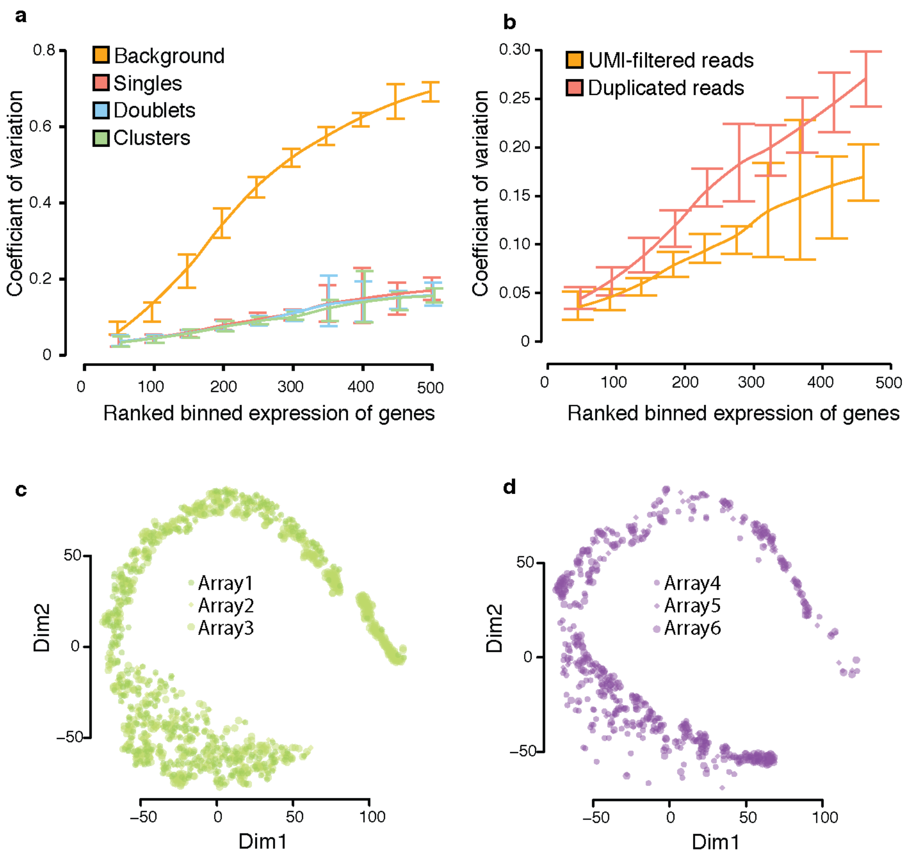

Supplementary Figure 2. MCF-7 as a model system. (a) Mean coefficient of variation as a function of ranked gene expression for all barcode groups. (b) Mean coefficients of variation of ranked gene expression for duplicated reads (red) and UMI-filtered reads (orange) showing noise reduction in the filtered reads. Error bars represent s. e. m. (c) *t*-SNE plot for all barcode groups produced on arrays 1-3. (d) *t*-SNE plot for all barcode groups produced on arrays 4-6. Arrays 1-3 were processed on one slide by the first operator, while arrays 4-6 were processed on another slide by a different operator.

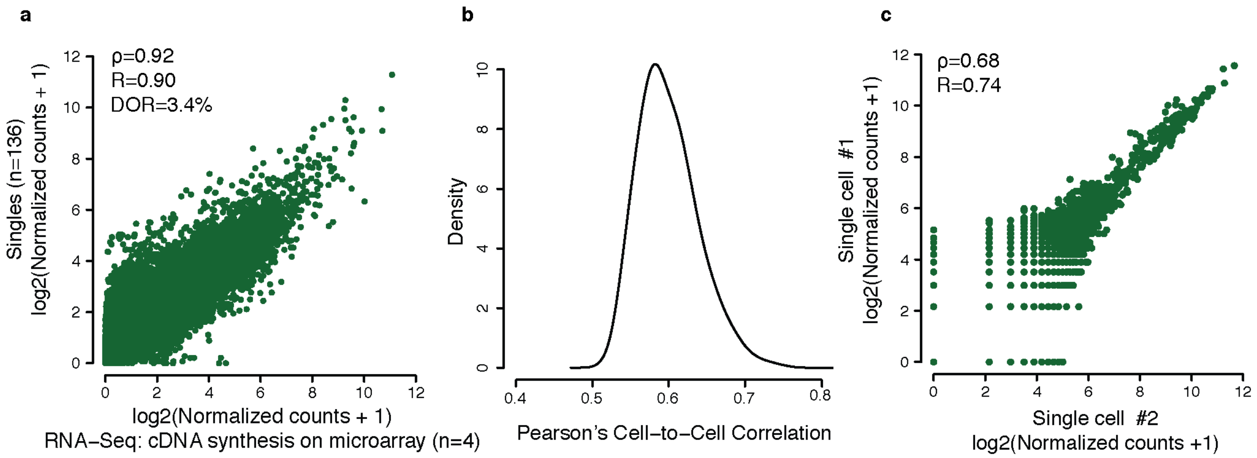

Supplementary Figure 3. Comparison of single-cell data. (a) Correlation plot between the single-cell average and RNA-sequencing (with cDNA synthesis performed on the barcoded microarray) data. (b) Density of Pearson's correlation between single cells. (c) An example correlation plot between two single cells.

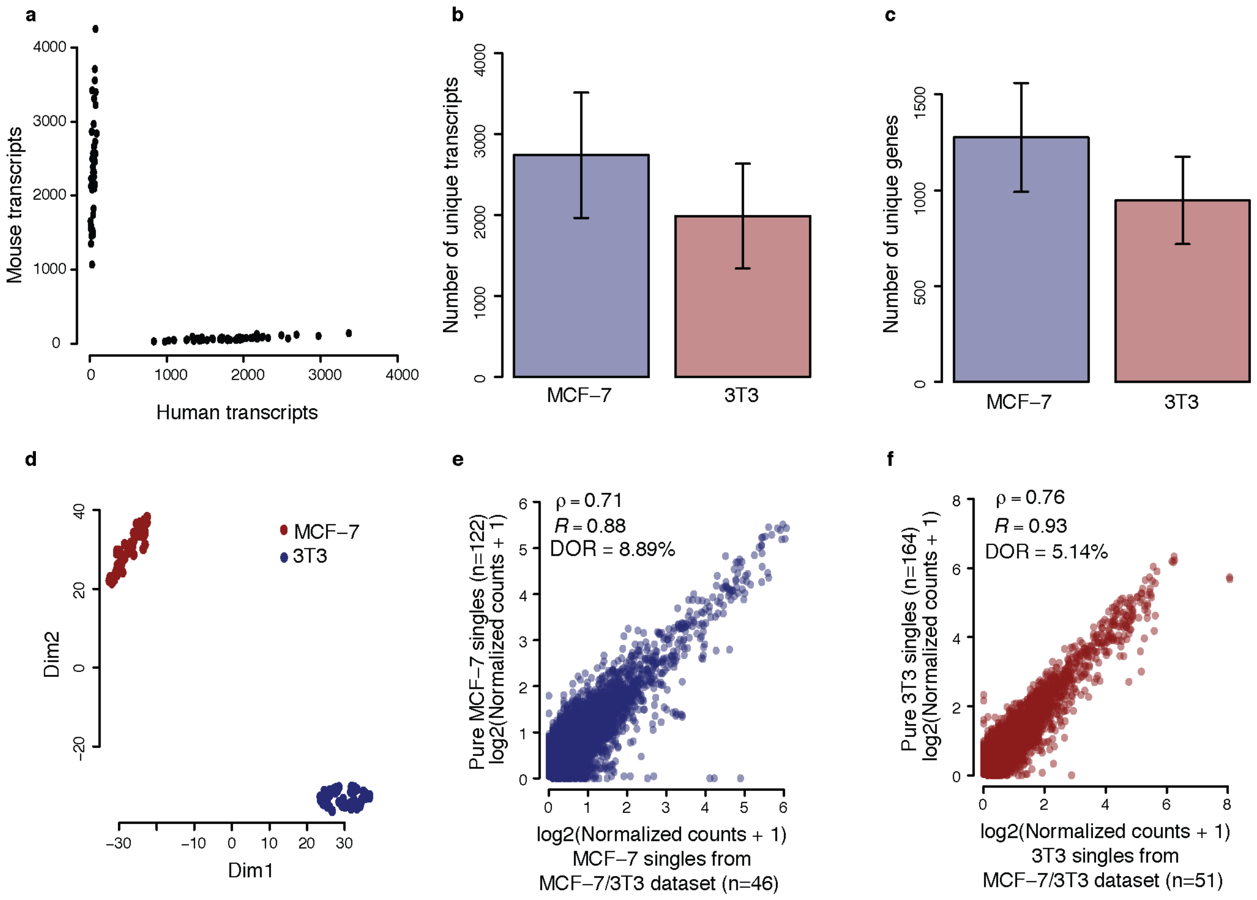

Supplementary Figure 4. Barcode crosstalk. (a) Scatterplot of species-specific reads per cell barcode. (b) Barplot showing numbers of transcripts per cell. Error bars represent s. e. m. (c) Barplot showing numbers of unique protein-coding genes per cell. Error bars represent s. e. m. (d) *t*-SNE plot showing separation of single cells based on orthologous species specificity. (e) Correlation plot between expression profiles of all single MCF-7 cells in the barcode crosstalk experiment and the experiment performed solely with MCF-7 cells. (f) Correlation plot between expression profiles of all single 3T3 cells in the barcode crosstalk experiment and the experiment performed solely with 3T3 cells.

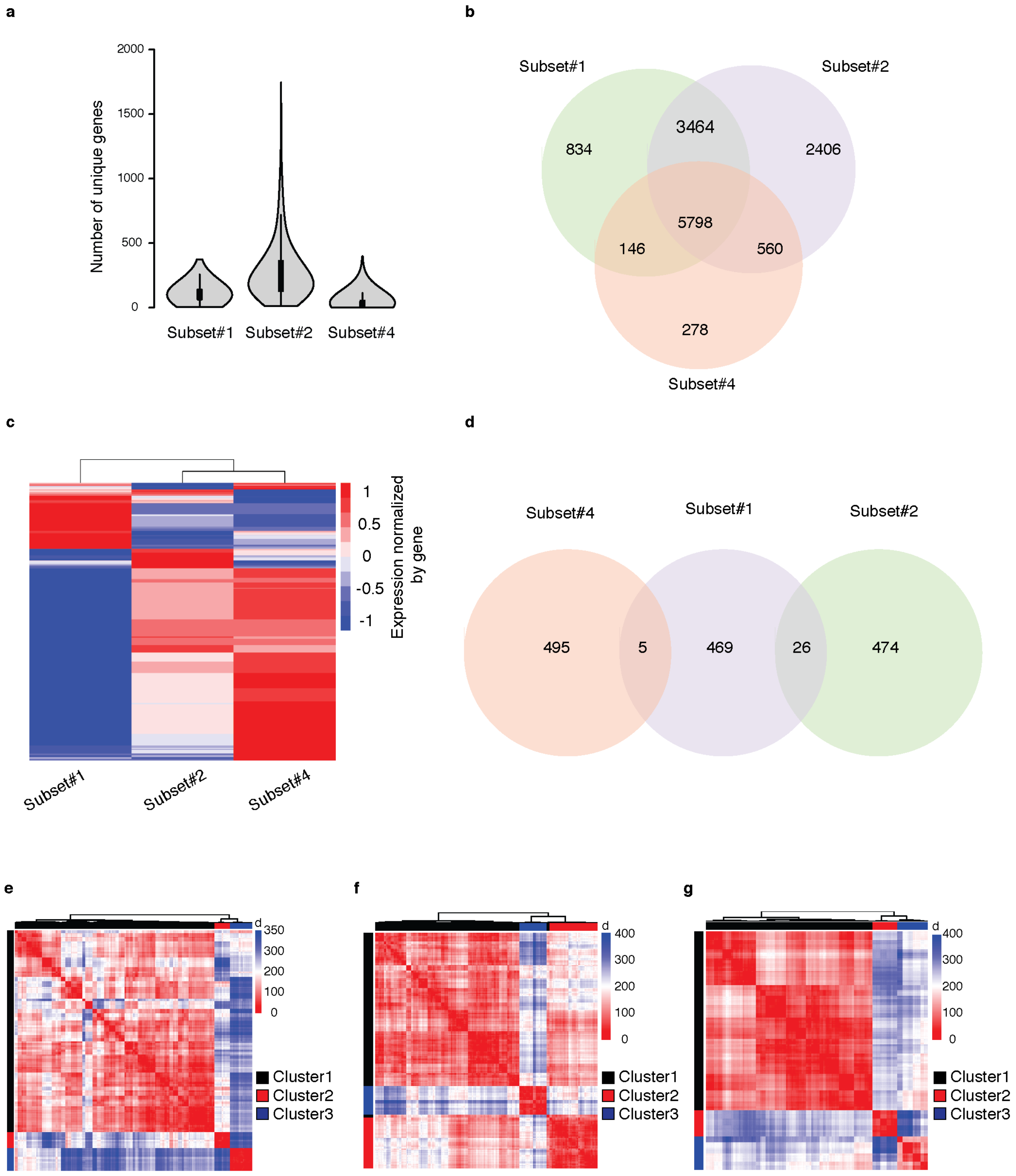

Supplementary Figure 5. Chronic lymphocytic leukemia cell data analysis. (a) Numbers of unique protein-coding genes expressed per cell. (b) Venn diagram showing numbers of shared genes per disease subset. (c) Heatmap of gene expression based on average expression of all

genes in each of the subsets. (d) Venn diagram showing overlaps of most highly abundant genes in each subset. (e-g) Dissimilarities heatmap and hierarchical clustering of single cells of subsets #1, #2 and #4 based on highly abundant genes, respectively.

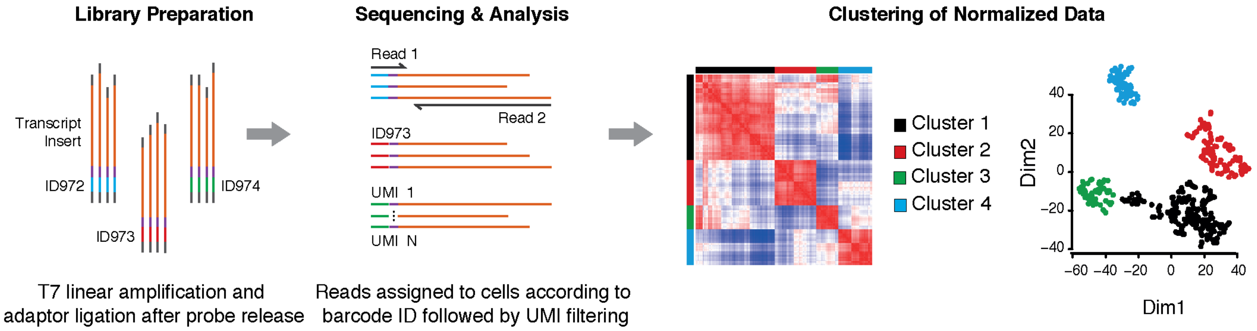

Supplementary Figure 6. Schematic illustration of data processing and analysis. Descriptive illustration of library preparation based on IVT, paired-end sequencing and data analysis including ID-demultiplexing, UMI-filtering and clustering.

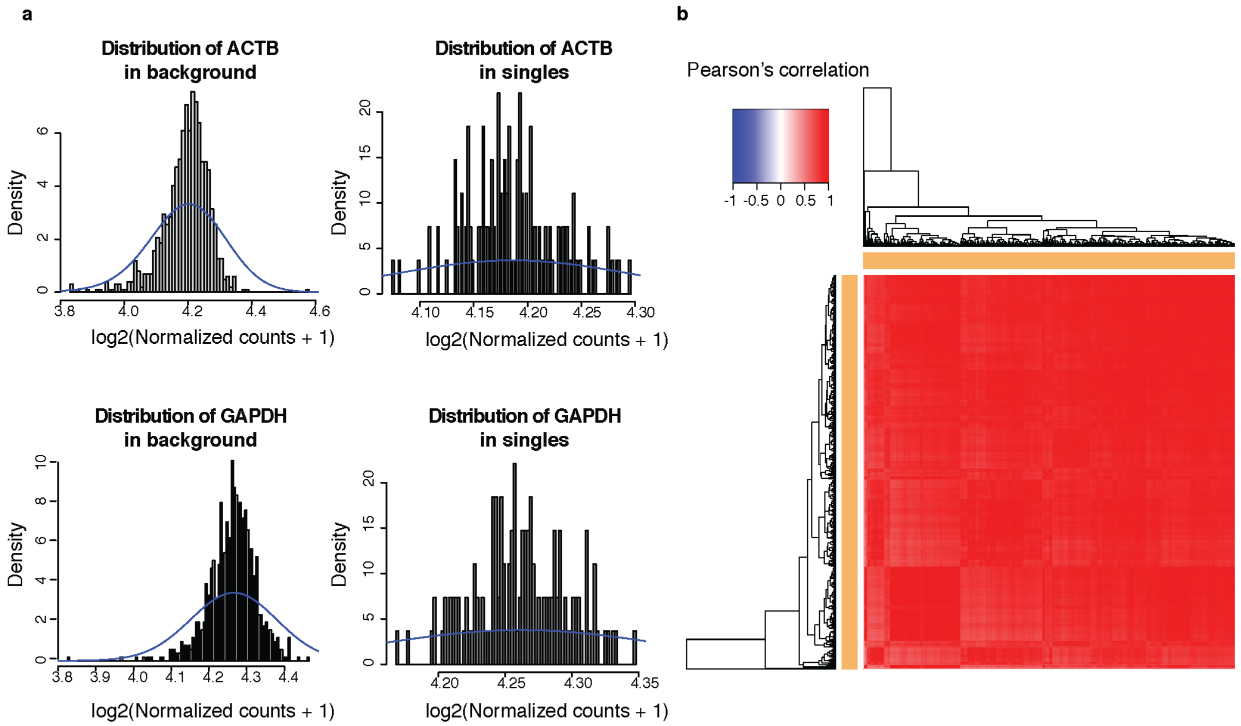

Supplementary Figure 7. Analysis of the background libraries. (a) Distribution of normalized reads in two example genes (ACTB and GAPDH) in the background and single-cell libraries. (b) Heatmap and hierarchical clustering of Pearson's correlation in the background libraries.

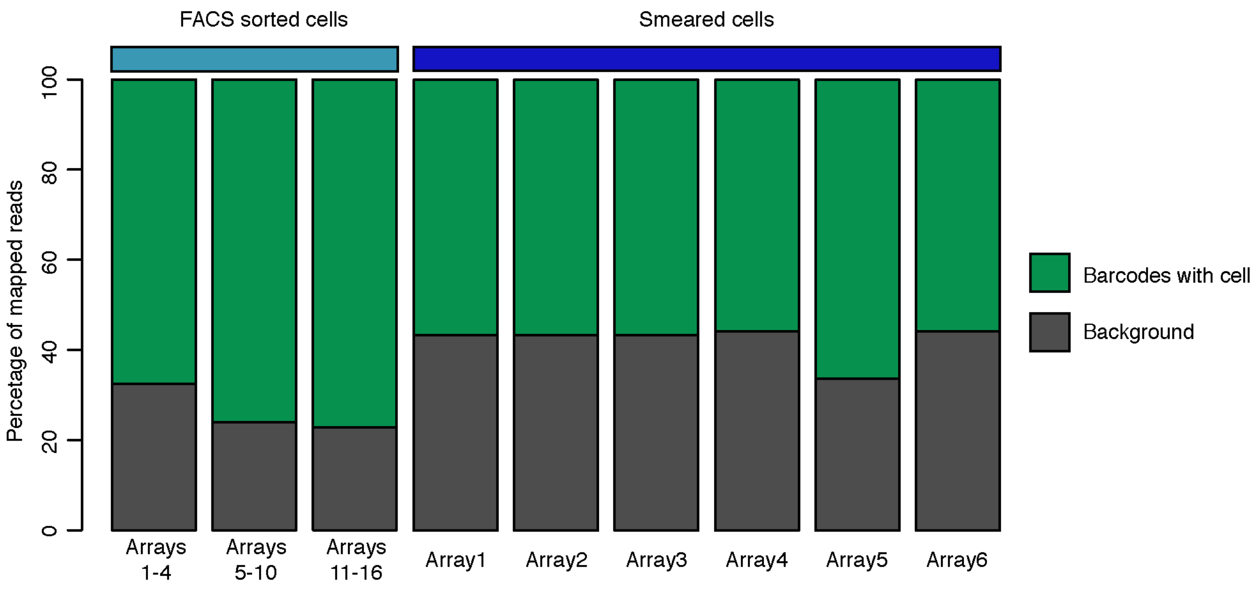

Supplementary Figure 8. Analysis of the background signal in FACS sorted and smeared libraries.

Supplementary methods

Array visualization

Complementary frame DNA primer with a 5' biotin modification (IDT, Leuven. Belgium) was coupled to streptavidin beads (Dynabeads Myone Streptavidin T1, Invitrogen, Life Technologies, Paisley, UK) using a previously described modified protocol[1,2]. The DNA primer and the streptavidin beads were mixed to a final ratio of 1200 primer molecules per bead. The reaction mixture was supplemented with 270 μL of the 2x B&W buffer (Bind and Wash buffer, 10 mM Tris-HCl at pH 7.5 with 1 mM EDTA and 2 M NaCl) and incubated for 7 min at room temperature. The beads were washed on a magnetic rack with 400 μL 1x TE (Tris-EDTA) buffer. Finally, the beads were resuspended in 400 μL 1x PBS-T (phosphate buffered saline with 0.05% Tween-20). The primer-coupled beads could be stored at 4 °C for at least 2 months. The microarray glass slide was attached to an ArrayIT (Sunnyvale, CA) hybridization cassette. 50 μL of the primer-bead suspension was added per microarray. A strong magnet was placed under the glass slide for 3 s. This was repeated five times. The magnet was then placed above the glass slide and the procedure was repeated. The primer-bead suspension was then slowly pipetted out and the slide was washed in prewarmed 2x SSC (saline sodium citrate) supplemented with 0.1% sodium dodecyl sulphate at 50 °C for 10 min, followed by 0.2x SSC and 0.1x SSC for 1 min at room temperature. The slides were left to dry at room temperature and stored in a low-humidity chamber (RH < 30%) until used for experiments.

Hematoxylin staining

To visualize the cells in bright field on the microarray glass slide, they were stained with Mayer's hematoxylin (Lilie's modification provided by Dako, Agilent Technologies, Santa Clara, CA, USA) for 4 min. The slide was then washed briefly in water and bluing buffer (also

from Dako) for 2 min to adjust the alkalinity of the sample. Again, the glass slide was washed briefly in water and dried at 37 °C for 5 min. The slides were mounted with 85% glycerol before imaging.

Fluorescent cell staining

Cells were stained for 10 min at room temperature after attachment to the microarray glass slide with DAPI (Molecular Probes, Life Technologies, Paisley, UK, diluted 1:2000 in 1x PBS) and CellTracker Deep Red Dye (Molecular Probes, Life Technologies, Paisley, UK, diluted 1:2000 in 1x PBS). The surface was washed once with 100 ul 1x PBS and left to air-dry. The slides were mounted with SlowFade Gold Antifade moutant (Invitrogen, Life Technologies, Paisley, UK) before imaging.

Cell staining for FACS

The patient samples were thawed in a water bath at 37 °C, then the cells were transferred to a 15 mL Falcon tube and 4 mL RPMI 1640 medium (Gibco, Life Technologies, Paisley, UK) was slowly added. The cell suspension was then centrifuged for 5 min at 1000 rpm. The supernatant was discarded and 5 mL 1x PBS was added. The cells were centrifuged again, the supernatant was discarded and the cell pellet was resuspended in 500 μL of 1x PBS.

To set the FACS gates, 5 μL of each cell suspension (except the negative control) was labelled with each of the staining antibodies at 1:100 dilution. The cells were stained with the anti-CD5 (FITC, clone UCHT2, Miltenyi Biotec, Lund, Sweden) and anti-CD19 (APC, clone LT19, Miltenyi Biotec, Lund, Sweden) markers together with the Hoechst nuclear stain (Life

Technologies, Paisley, UK) at 1:50 ratio. The remaining cell suspension was labelled with the antibodies at a 1:33 ratio and the Hoechst nuclear stain at a 1:50 ratio.

This was followed by a 30 min incubation at 4 °C in the dark. The cells were then washed in 1x PBS and centrifuged for 5 min at 1000 rpm. The supernatant was removed and the cells were fixed in 75 µL of 2% formaldehyde for 8 min at room temperature. To remove the remaining fixative, cells were centrifuged for 5 min at 1000 rpm, then resuspended in 500 µL of 1x PBS.

Bulk RNA-seq library preparation - cDNA synthesis in solution

Libraries were prepared from total extracted MCF-7 RNA material using six pooled barcoded DNA oligonucleotides (not printed on the array surface) at a final concentration of 3 µM complemented with 1.25 µM dNTPs. The starting material was 300 ng total fragmented RNA. Before starting cDNA synthesis, the mix was heated to 65 ºC, cooled, then cDNA synthesis was initiated by adding 1x FS buffer, 2.5 mM DTT, 10U RNaseOUT and 100U Superscript II Reverse Transcriptase (Invitrogen, Life Technologies, Paisley, UK). A TSO (5-ACACGACGCTCTTCCGATCTNNNNNNNNATrGrGG-3) was added to the reaction at 5 µM final concentration. The mixture was incubated at 42 ºC for 1 h and 70 ºC for 15 min. The resulting cDNA was purified using the Agencourt RNAClean XP system (Beckman Coulter, Pasadena, CA, USA) according to the manufacturer's instructions and eluted in 10 µL water. An indexing PCR reaction was performed as previously described. These libraries were prepared in duplicate. To prepare reference carrier material, total reference human brain RNA (Agilent, Technologies, Santa Clara, USA) was again fragmented to an average length of 200 nt and cDNA was synthesized as described in this subsection, starting with 1 µg of the fragmented total RNA.

Bulk RNA-seq library preparation - cDNA synthesis on the array surface

Total MCF-7 RNA (300 ng) was fragmented and the material was added directly to the array during the cDNA synthesis. This step and all of the following library preparation steps were performed as already described. These libraries were prepared in quadruplicate. A ERCC spike-in control mix (Ambion, Life Technologies, Paisley, UK) was added (1:100 as recommended by the manufacturer) to 1 μg total A549 total RNA before fragmentation and fragmented as described above. The material was added directly to the array during cDNA synthesis. This step and all of the following library preparation steps were performed as already described. These libraries were also prepared in quadruplicate.

CEL-Seq library preparation

CEL-seq barcodes were exchanged for MASC-seq barcodes (single barcode per well) in order to utilize UMI-filtering. 48 Single MCF-7 DAPI-stained cells were sorted in a 96-well skirted PCR plate pre-aliquoted with the barcode primer and water to a total volume of 1.2 μl. The samples were immediately frozen to -80 ºC until processing. The libraries were prepared according to the CEl-seq protocol[11]. After cDNA synthesis the cells were pooled and 3 ng carrier material added as recommended. An extra cleaning step had to be performed after the cDNA synthesis and adaptor ligation steps due to too high salt concentration and otherwise incorrect adapter incorporation that prevented efficient library clustering. The libraries were indexed as described previously with the MASC-seq system.

Adding barcoded reference carrier material

Before starting the library preparation, all cell-based samples were spiked with barcoded RNA reference cDNA material created as described in the previous section, to ensure there was enough material during the IVT (*in vitro* transcription) reaction. To ensure that the carrier material did not dominate in the sequencing step, qPCR was performed using a GAPDH primer pair to determine the cDNA amounts present in both the patient samples and the barcoded reference material.

Examining array print quality

To establish exact printed positions of all of the barcodes including the frame, Cy3-labeled complementary DNA oligonucleotides were hybridized to the surface after probe release. The Cy3 print of the entire array could be subsequently overlaid with the hematoxylin images and used in the data processing to ensure correct decoding of the cell-barcode combinations.

Supplementary References

[1] Pettersson, E., Ahmadian, A., & Ståhl, P. L. A Novel Method for Rapid Hybridization of DNA to a Solid Support. *PLoS ONE* **8**, e70504 (2013).

[2] Ståhl, P. L., *et al.* Visual DNA - Identification of DNA sequence variations by bead trapping. *Genomics* **90**, 741–745 (2007).
